# There are no equal opportunity infectors: Epidemiological modelers must rethink our approach to inequality in infection risk

**DOI:** 10.1371/journal.pcbi.1009795

**Published:** 2022-02-09

**Authors:** Jon Zelner, Nina B. Masters, Ramya Naraharisetti, Sanyu A. Mojola, Merlin Chowkwanyun, Ryan Malosh

**Affiliations:** 1 Dept. of Epidemiology, University of Michigan School of Public Health, Ann Arbor, Michigan, United States of America; 2 Center for Social Epidemiology and Population Health, University of Michigan School of Public Health, Ann Arbor, Michigan, United States of America; 3 Dept. of Sociology, School of Public and International Affairs & Office of Population Research, Princeton University, Princeton, New Jersey, United States of America; 4 Dept. of Sociomedical Sciences, Mailman School of Public Health, Columbia University, New York, New York, United States of America; University of Virginia, UNITED STATES

## Abstract

Mathematical models have come to play a key role in global pandemic preparedness and outbreak response: helping to plan for disease burden, hospital capacity, and inform nonpharmaceutical interventions. Such models have played a pivotal role in the COVID-19 pandemic, with transmission models—and, by consequence, modelers—guiding global, national, and local responses to SARS-CoV-2. However, these models have largely not accounted for the social and structural factors, which lead to socioeconomic, racial, and geographic health disparities. In this piece, we raise and attempt to clarify several questions relating to this important gap in the research and practice of infectious disease modeling: Why do epidemiologic models of emerging infections typically ignore known structural drivers of disparate health outcomes? What have been the consequences of a framework focused primarily on aggregate outcomes on infection equity? What should be done to develop a more holistic approach to modeling-based decision-making during pandemics? In this review, we evaluate potential historical and political explanations for the exclusion of drivers of disparity in infectious disease models for emerging infections, which have often been characterized as “equal opportunity infectors” despite ample evidence to the contrary. We look to examples from other disease systems (HIV, STIs) and successes in including social inequity in models of acute infection transmission as a blueprint for how social connections, environmental, and structural factors can be integrated into a coherent, rigorous, and interpretable modeling framework. We conclude by outlining principles to guide modeling of emerging infections in ways that represent the causes of inequity in infection as central rather than peripheral mechanisms.

## Introduction

In March 2020, a prescient news item in *Science* proclaimed that infectious disease transmission models had taken on “life or death importance” [1] as tools in the fight against Severe Acute Respiratory Syndrome Coronavirus 2 (SARS-CoV-2). Despite the pivotal role they have played, most mechanistic models used to guide the global response to SARS-CoV-2 paid little direct attention to the causes of the massive socioeconomic and racial inequities that have characterized the pandemic in the United States and around the world [2–7]. This reflects the absence of a theoretical and methodological framework needed to deploy equity-oriented models with the same speed and rigor as those focused on understanding and forecasting population-level outcomes.

The challenges of maintaining equity and minimizing population-level risks in the face of an emerging pathogen—particularly in highly unequal societies such as the US—has forced infectious disease modelers to grapple with how transmission models can and should account for social factors going forward [8,9]. Much of this work has focused on the use of detailed information on mobility and individual behavior to make better predictions of epidemic trajectories and estimates of model parameters. In broad strokes, they argue for more detailed data collection and closer partnership with communities to ensure that models incorporate local data and address real-world needs. While recent and ongoing innovations in the collection and analysis of high-resolution social network and mobility data [10] could be powerful tools for highlighting and addressing infection inequality, this outcome is not guaranteed over the long term. This piece explains why.

Here, we bridge key theoretical ideas from social epidemiology and infectious disease transmission modeling. We plot a path forward in which innovation in the ability of models to address inequity occurs in parallel—and at an equal pace—with other leaps forward in data collection and analysis. We draw on examples most relevant to the US; however, the framework articulated here can and should be extended to examine both global, between-country inequities, as well as power relationships and social inequalities within low- and middle-income countries (LMICs) and other wealthy countries. We advocate for an approach to infectious disease modeling that explicitly includes overarching social factors, like socioeconomic status (SES) [11], racism [12], segregation [13], and disability-, age-, and sexuality-related stigma [14]—which put individuals and populations “at risk of risk” [15]. In doing this, we draw on a wide and deep literature in social epidemiology, the social sciences, and applied statistics that has been deeply engaged with questions about the causal roles played by economic inequality, racism, gender, and sexuality on health outcomes for decades. We do not in any way eschew the use of detailed, high-resolution social data as a tool for combating infectious disease. Instead, we worry that the power of these tools to prevent and ameliorate inequity may be squandered if their use is not informed by key concepts from the vast literature on health disparities in both infectious and noncommunicable disease. In turn, when social-structural causes are excluded from transmission models, they cannot be used to examine how structural remedies, like wealth transfers, universal healthcare, labor protections, antidiscrimination policy, and guaranteed housing might impact incidence and mortality rates. Finally, our arguments in favor of this integration would be meaningless if mathematical models were not themselves such powerful tools for addressing theoretical and empirical questions within and outside of infectious disease epidemiology [16], and if we did not believe that the types of models developed by infectious disease epidemiologists could also help to improve the state of the art in social epidemiology and the social sciences [17,18].

### Understanding why equity has been left out of many models may show how to bring it back in

The small number of models that directly account for inequities in infection reflects the lack of a digestible framework for including sociostructural inequality as a first-class feature of transmission models. The current moment provides an opportunity to close this gap. Coronavirus Disease 2019 (COVID-19) has increased awareness of the fact that flesh-and-blood social inequities underlie the values of abstract model parameters. For example, Richardson and colleagues have argued that, in the US, the basic reproduction number, *R*_0_, must be understood not only in terms of pathogen biology and individual behavior, but also of racialized structural violence flowing from the legacy of slavery, which compels some to be exposed and allows others to remain safe [19]. Similarly, the social epidemiologist David Williams has argued that herd immunity should be reconceptualized in explicitly social terms, which recognize that the level of immunity from natural infection and vaccination is a function not only of pathogen biology, but of the social and economic systems that propel transmission and health behavior [20].

While structural inequity has been largely absent from models of acute respiratory infections (ARIs) such as SARS-CoV-2 and pandemic influenza, it has long been at the heart of modeling work on HIV as well as a range of viral and bacterial sexually transmitted infections (STIs). Because transmission of STIs is so closely related to interpersonal relationships and sexual behaviors that have been highly stigmatized, the sociostructural factors that shape STI and HIV transmission have been harder to ignore than for viral respiratory infections. As a result, there is a vast literature examining inequities in HIV and STI infection using population- and network-based transmission models [21–23]. By contrast, transmission of ARIs through the air via respiratory droplets and aerosols makes the mapping of social relationships onto transmission appear less straightforward. This is despite the fact that close relationships, such as those among family members and cohabitants, are key modes of SARS-CoV-2 transmission [24,25], and smaller-scale studies have demonstrated the role of individual-level patterns of contact in influenza transmission [26]. This disconnect historically contributed to the erroneous, but pervasive, idea that ARIs are “equal opportunity infectors” [27] for which the structure of social networks and systems of global and domestic inequality and oppression are less important than for quintessentially social pathogens like HIV, STIs, and tuberculosis (TB). COVID-19 has dealt a severe blow to this idea, with key models directly integrating social network [28] and mobility data [29], in some cases with infection inequality squarely in the crosshairs [30]. But it remains to be seen how we can capitalize on this momentum to make the necessary, long-term changes to the modeling toolkit.

Doing this is critical, because the way we represent cause and effect in transmission models has enormous implications for policy and practice. Transmission models let practitioners, policymakers, and researchers envision alternative futures that might be realized through intervention, policy, and social action. When these models exclude sociostructural factors that drive inequity—income, education, racial residential segregation—they preclude the ability to explore the possibility of structural change as an epidemiological tool on par with nonpharmaceutical interventions (NPIs), vaccination, and testing. In the following sections, we outline an “equity-forward” approach to transmission modeling that places the fundamental sociostructural causes of infection inequality on an equal level with the biological and behavioral features of transmission. Our goal is to articulate a vision of socially informed modeling that is squarely focused on understanding how imbalances in social power drive infection inequalities and suggesting social and political remedies to these disparities. This work is inspired by the challenge laid out by the operations researcher Edward Kaplan who wrote that “the world is full of problems, but one has to work to structure them as such.” [31] What follows represents an attempt to define the problem of social inequity in infection outcomes in terms that align with the structure of infectious disease transmission models.

### What are the goals of equity-forward transmission modeling?

Transmission models are often used to answer “what-if” questions from a perspective of authority: What will happen if governments impose quarantines or mask-wearing orders? How should national, state, or local public health authorities allocate scarce vaccines or target outreach efforts to increase uptake? These questions lend themselves to a focus on predicting the timing and spatial distribution of infection over short and long time horizons as a function of a set of potential interventions that are proximal to individual-to-individual transmission, such as mask-wearing, social distancing, testing, and vaccination. This type of modeling is most relevant for informing a decision-maker who is anticipating, planning, and responding to events in the near future [32], but may obscure the role of higher-order social structures in enabling or constraining the ability of these types of “downstream” interventions to have the desired effect [2]. Furthermore, it presupposes that the decisions of policymakers and other authorities are the most important determinants of disease outcomes, potentially contributing to the obscuring of sociostructural determinants as well as the ability of more bottom-up social movements to directly impact infection outcomes.

For infectious disease models to be useful tools for addressing inequity, in addition to being predictive or prognostic in nature, they also need to be *diagnostic* and *forensic* tools that can characterize the causes of disparity in disease outcomes. Because the remedies to sociostructural inequities are not discrete, one-off interventions, but instead messy and protracted contests over power, equity-forward models must provide evidence and ideas that can propel and support efforts at social and political change.

Rather than a primary focus on predicting aggregate, population-level patterns (i.e., the pace and timing of infection), an equity-forward modeling approach should be concerned with characterizing *who* is likely to be infected and how the distribution of infection reflects allocations of economic and social power at the population level. Accomplishing this requires models that simultaneously accommodate social and biological mechanisms of interpersonal dependence at levels higher than individual-to-individual interactions. For example, aggressive COVID-19 lockdowns were enabled by the labor of healthcare, retail, delivery, and warehouse workers who continued to provide goods and services to people who were able to remain at home. As a result of economic exploitation and inadequate workplace safety, these workers bore much of the brunt of early exposure, infection, and death. This means that lower rates of exposure experienced by wealthier individuals and whites [33] resulted directly from economic and racialized power imbalances. This pattern, dubbed the “inverse interdependent welfare principle” by the sociologist Erik Olin Wright [34], has been applied to many problems in social epidemiology [35] and has clear implications for understanding how policies and social action that shift basic power dynamics can impact infection risks. For transmission models to be faithful representations of the way infection risk occurs, characterizing this type of sociostructural dependence needs to be treated with equal importance as faithfully representing the rate of infection and the transitions between biological states an individual experiences following infection.

### Equity-forward models must address theoretical and applied questions

For transmission models to be effective in attacking infection inequalities, they must mechanistically link social causes with biological outcomes. Doing this credibly necessitates developing relatively abstract models that let us explore basic questions around social causation, in addition to more concrete ones that can guide policy and social action using real-world data. Theoretical models are essential for addressing questions about measurement of the effects of the types of layered interventions associated with social policy measures on infection outcomes [36]: For example, comprehensive housing reform—clearly an issue related to risks of infection [37–39] and inequity in infection outcomes [40]—includes financial tools like housing vouchers, regulatory changes that provide enhanced protections against eviction and foreclosure to renters and homeowners, and zoning modifications to allow for multifamily home construction in residential areas, to name a few.

Beyond differences in the modality of intervention, the thoroughness of implementation of social policies is likely to vary across jurisdictions, and the impact of financial assistance to renters will vary as a function of local housing market conditions. And just as theoretical models have been critical for pushing the science of infectious disease epidemiology and ecology forward by yielding insights into the population-level impacts of superspreading [41], the role of birthrates and seasonal forcing in the transmission dynamics of vaccine-preventable diseases [42], and the reflection of transmission in pathogen genomes [43], among many other key questions, similar theoretical exploration is essential for building the foundations that more-complex equity-forward models can be built upon.

However, to be useful adjuncts to day-to-day social and political action, models and modelers also need to engage with concrete questions that relate to the types of crises that characterize emerging infections from Ebola to SARS-CoV-2 in the present era. For example, the burden of COVID-19 infection and mortality in jails, prisons, and immigration detention facilities in the US has resulted in modeling studies focused on the potential impact of decarceration on the risk to incarcerated individuals and their communities [44,45]. But this focus can be broadened to examine the impact of other specific legislative and administrative interventions on infectious disease transmission and infection inequities. For example, Nande and colleagues used counterfactual simulation to estimate the number of COVID-19 cases prevented by the Centers for Disease Control and Prevention’s (CDC’s) eviction moratorium during Fall 2020 [46]. Such work is critical for building short- and long-term support for social and political actions aimed at preventing the uptick in deaths associated with ending these protections [47].

### Defining the key mechanisms and outcomes in an *equity-forward* transmission model

We advocate for an adaptation of the *fundamental-cause (FC)* perspective on health inequity to the problem of transmission. Link and Phelan define a fundamental social cause of health inequity as a factor, like SES or racism [13], that puts individuals and populations “at risk of risks” [15]. The FC approach focuses on how differentials in social power impact access to the material and social resources—money, occupation, housing, medical care, education, prestige—that structure risks of infection and death. There is nothing new in the idea that social factors are causes of infection on par with biological ones. In the 1950s, the social medicine pioneers René and Jean-Baptiste Dubos referred to the *Mycobacterium tuberculosis* as a necessary, but insufficient, precondition for TB infection, with social and occupational factors ultimately shaping exposure and susceptibility [48,49]. Similarly, public health historian Samuel Kelton Roberts detailed how racial residential segregation drove TB infection and mortality among African-Americans in 20th century Baltimore via impacts on housing, workplace conditions, medical treatment, and public health policies [50]. These mechanisms have been repeatedly articulated in narrative histories, risk-factor analyses, and mixed-methods studies of many infections including cholera [51], HIV [52,53], and malaria [54,55]. Clouston and colleagues found that while high-income US counties were the first to see an introduction of SARS-CoV-2 infection, the pace of infection and mortality in these counties quickly slowed through NPIs (e.g., work from home, school closures). Meanwhile, rates of infection and death exploded in counties with lower-income and higher proportions of non-white residents where NPIs were less feasible [2]. Given the ubiquity of the FC perspective in explicit and implicit understandings of infectious disease risk, its absence from transmission modeling is surprising.

We argue that the FC approach provides a useful set of principles that can be used to guide both the goals and data collection necessary to build the foundations of equity-forward transmission modeling. To conclude this essay, we outline 3 of the core concepts underlying FC theory and highlight how they relate to the mechanisms of infectious disease transmission, with an eye toward how they can be integrated into transmission models:

## 1. Social factors such as SES and racism are fundamental causes of infection because they operate on *multiple* intervening mechanisms that drive transmission, including housing, occupation, healthcare, and others

**Fig 1:**
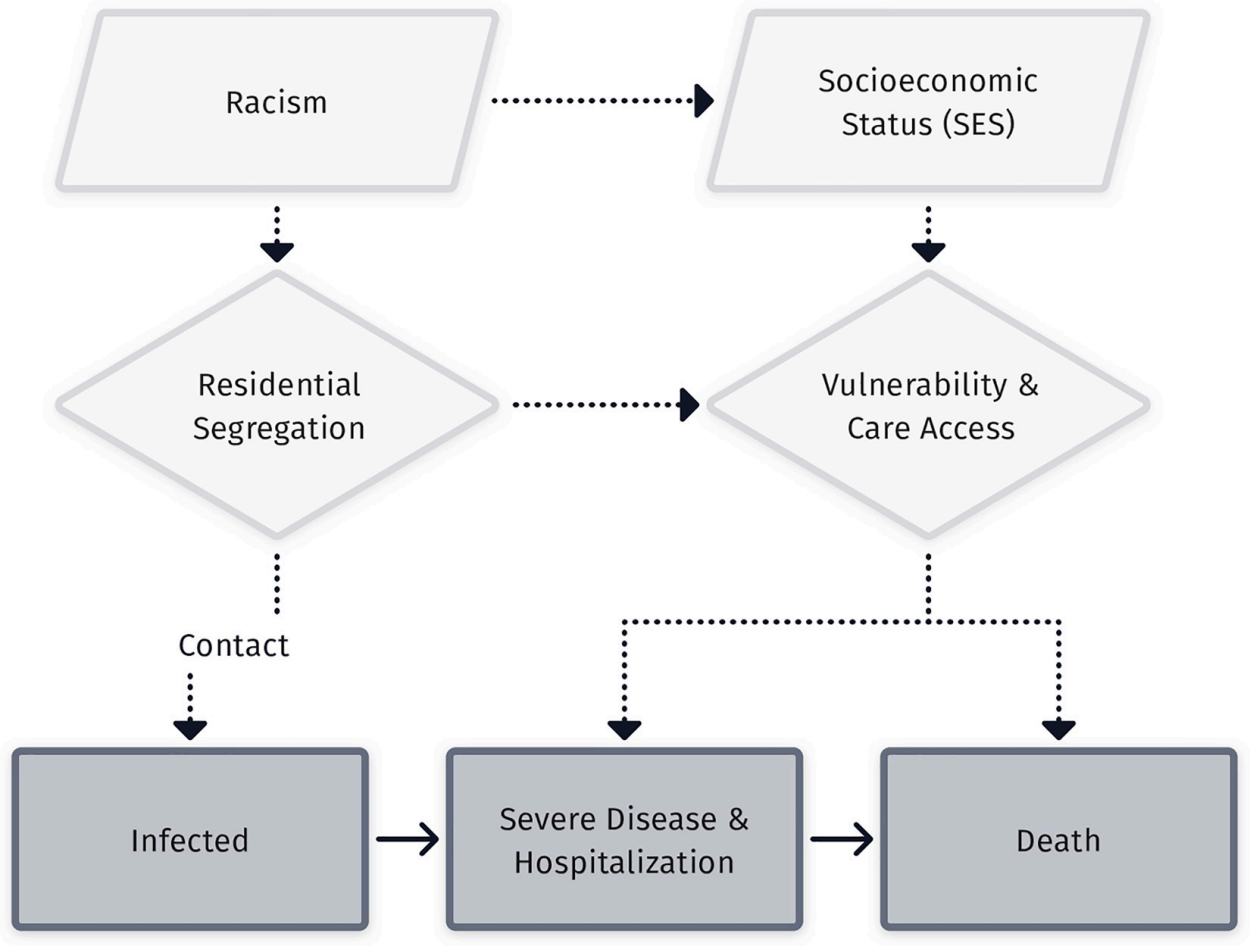
Illustration of the impact of fundamental causes on inequity in infection through multiple intervening mechanisms and multiple outcomes. The figure illustrates key relationships between high-level fundamental causes of social inequality (parallelograms) on risks of infection and disease progression (rectangles) via their impact on more-proximal risks for exposure, severe disease upon infection and death (diamonds). Solid lines represent flows between disease states, while dotted lines illustrate relationships between risk factors and their impacts on susceptibility to infection acquisition and the rate of progression through escalating phases of disease severity. For visual clarity, only a subset of potential relationships is illustrated. For example, racism impacts vulnerability and access to care directly as well as indirectly, and SES and wealth often contributes to residential segregation. SES, socioeconomic status.

The proximal factors that drive exposure and mortality risk are socially correlated: Individuals in high-risk occupations are more likely to live in crowded conditions and have poor access to acute and preventive care. Due to such racial residential and occupational segregation, individuals sharing these risks are also more likely to have high rates of contact with each other, concentrating the impact of these differential risks within marginalized groups [56]. In Fig 1, we illustrate the way high-level sociostructural determinants, like SES and racism, drive disparities in infection outcomes though their impact on multiple intervening mechanisms. *Transmission models must not ignore the fact that these proximal drivers flow from upstream causes*, *lest they may make overly optimistic projections of the impact of tweaks to individual proximal risk factors and underestimate the impact of higher-level social interventions that would improve multiple downstream factors simultaneously*.

## 2. Protective NPIs, policies, and medical innovations reach more-advantaged individuals first, and this access is the cause of deprivation among lower-SES individuals and communities

The COVID-19 pandemic has exposed how NPIs such as social distancing are structured by economic and racial advantage. In the context of an emerging infection, these effects may be even more acute than with many noncommunicable diseases: While more-advantaged individuals wait out infection at home, the response evolves, clinical management of infection improves, and case-fatality rates fall, allowing these groups to sidestep the worst effects almost entirely, while minoritized and poorer groups take the brunt of infection and death [4,57]. *Including mechanisms of social and economic dependence in exposure*, *i*.*e*., *the ways in which one group’s increased exposure facilitates the decreased exposure of another is essential for transmission models to be useful tools for identifying and mitigating inequity*.

## 3. The same sociostructural factors drive inequity across *multiple infectious disease outcomes*

The sociostructural factors that drive risks for one pathogen are likely to influence others, resulting in *syndemics* of infection [58,59]. The immediate toll of COVID-19 mortality has disproportionately affected low-SES and minority communities. However, emerging evidence suggests the risk of a “double jeopardy” effect: Those communities where hospital systems were overwhelmed, already-insufficient primary care fell behind, and where children were unable to keep up with routine immunizations, are now at risk of outbreaks of other vaccine-preventable diseases, such as measles, pertussis, and others. In addition, risk factors for COVID-19 strongly overlap with those for infections such as tuberculosis, influenza, fungal infections such as coccidiomycosis [31], HIV/STIs, and others. Vaccine hesitancy and poor access to prevention and care also impact risk for multiple pathogens, and a higher prevalence of comorbid noncommunicable diseases and coinfections dramatically increases risks for poor outcomes including hospitalization and death. *Consequently*, *models that account for potential “knock-on” effects of one set of social causes on multiple disease outcomes—how the risks for SARS-CoV-2 are related to risks for influenza*, *other ARIs*, *HIV*, *and other infections—are critically necessary to assess the full scope of damage associated with upstream inequities and to anticipate emergent disease inequalities*.

## Conclusions

Deepening the integration between sociostructural and biological mechanisms in transmission models is an urgent necessity. The challenge of this undertaking should not be understated [60], nor should the substantial contributions of transmission modelers during the COVID-19 pandemic. We echo the argument made by Bertozzi and colleagues in the early days of the pandemic [61]: Rather than stumbling over attempts at hyperrealism, transmission models should focus on characterizing broad trends in inequity, the mechanisms that generate them, and multilevel interventions that might work to ameliorate infection inequities. We also should not pursue a single “correct” model that includes all these mechanisms and outcomes at once. Instead, equity-forward models should be another element of the epidemiological toolkit, alongside their forecasting and predictive counterparts.

The value of these models comes from their potential to force policymakers, practitioners, and the public to envision alternative futures in which infection inequality is both easy to anticipate and possible to prevent. We hope that models that include these mechanisms can be tools for achieving the social herd immunity described by Williams and Cooper [20], but getting there will require a sober reckoning of how far we currently are from it. Without models that simultaneously speak the languages of transmission, evolution, and social stratification, sociostructural changes that come from contesting power will once again be left out of the universe of possibilities that can be explored using transmission models. Including these mechanisms does not guarantee a better outcome in the next crisis, but it does position models—and modelers—to be ready to address questions of health justice early and often in the next crisis.
